# Post-Traumatic Stress Disorder and other mental disorders in the general population after Lorca’s earthquakes, 2011 (Murcia, Spain): A cross-sectional study

**DOI:** 10.1371/journal.pone.0179690

**Published:** 2017-07-19

**Authors:** Fernando Navarro-Mateu, Diego Salmerón, Gemma Vilagut, Mª José Tormo, Guadalupe Ruíz-Merino, Teresa Escámez, Javier Júdez, Salvador Martínez, Karestan C. Koenen, Carmen Navarro, Jordi Alonso, Ronald C. Kessler

**Affiliations:** 1 Unidad de Docencia, Investigación y Formación en Salud Mental (UDIF-SM). Servicio Murciano de Salud. Departamento de Psicología Básica y Metodología. Universidad de Murcia. Murcia. Spain; 2 IMIB-Arrixaca. Murcia. Spain; 3 CIBER de Epidemiología y Salud Pública (CIBERESP). Madrid. Spain; 4 Servicio de Epidemiología. Consejería de Salud. Murcia. Spain; 5 Departamento de Ciencias Sociosanitarias. Universidad de Murcia. Murcia. Spain; 6 IMIM-Institut Hospital del Mar d´Investigacions Médiques. Barcelona. Spain; 7 Fundación para la Formación e Investigación Sanitarias (FFIS) de la Región de Murcia. Murcia. Spain; 8 IMIB BIOBANC-MUR. Biobanco-HUVA-AECC-FFIS. Murcia. Spain; 9 Sociedad Española de Patología Digestiva. Fundación Española del Aparato Digestivo. Madrid, Spain; 10 Instituto de Neurociencias. Alicante. Spain; 11 Department of Epidemiology. Harvard T.H. Chan School of Public Health. Boston, United States of America; 12 Departamento de Salud y Ciencias Experimentales. Universidad Pompeu Fabra. Barcelona. Spain; 13 Department of Health Care Policy. Harvard Medical School. Boston, United States of America; Stellenbosch University, SOUTH AFRICA

## Abstract

**Aims:**

To describe the prevalence and severity of mental disorders and to examine differences in risk among those with and without a lifetime history prior to a moderate magnitude earthquake that took place in Lorca (Murcia, Spain) at roughly the mid-point (on May 11, 2011) of the time interval in which a regional epidemiological survey was already being carried out (June 2010 –May 2012).

**Methods:**

The PEGASUS-Murcia project is a cross-sectional face-to-face interview survey of a representative sample of non-institutionalized adults in Murcia. Main outcome measures are prevalence and severity of anxiety, mood, impulse and substance disorders in the 12 months previous to the survey, assessed using the Composite International Diagnostic Interview (CIDI 3.0). Sociodemographic variables, prior history of any mental disorder and earthquake-related stressors were entered as independent variables in a logistic regression analysis.

**Findings:**

A total number of 412 participants (response rate: 71%) were interviewed. Significant differences in 12-month prevalence of mental disorders were found in Lorca compared to the rest of Murcia for any (12.8% vs 16.8%), PTSD (3.6% vs 0.5%) and other anxiety disorders (5.3% vs 9.2%) (p≤ 0.05 for all). No differences were found for 12-month prevalence of any mood or any substance disorder. The two major predictors for developing a 12-month post-earthquake mental disorder were a prior mental disorder and the level of exposure. Other risk factors included female sex and low-average income.

**Conclusions:**

PTSD and other mental disorders are commonly associated with earthquake disasters. Prior mental disorders and the level of exposure to the earthquakes are the most important for the development of a consequent mental disorder and this recognition may help to identify those individuals that may most benefit from specific therapeutic intervention.

## Background

Earthquakes are frequent all worldwide but most of them are inconsequential either because of their intensity or their distance from populated areas. Those resulting in significant loss of life are infrequent [1]. The Center for Research on the Epidemiology of Disasters (CRED) provides an updated list of disasters [2] and, for one to be considered as so, an earthquake has to fulfill one or more of the following criteria: i) ten or more people reported killed, ii) 100 or more people reported affected, iii) call for international assistance, or iv) declaration of a state of emergency. Since 2000, a total of 456 earthquake-related disasters have been documented globally (32 in Europe).

Only one Spanish earthquake since 2000 has fulfilled the above criteria that of May 11, 2011 in Lorca Southeastern Spain, a municipality of nearly 100,000 residents located 58 kilometers from the city of Murcia. It was of moderate magnitude (5.1 Mw) and was preceded by a somewhat smaller one (4.5 M_w_) and followed by almost 50 aftershocks of minor magnitude in the ensuing few days. The shallow depth of the epicenter (1 kilometer or 0.6 miles) increased the devastating impact, resulting in nine deaths, more than 300 injuries, and many more left homeless. Nearly 80% of properties in the area suffered some damage and many in the worst affected areas were demolished in the following months. A high prevalence of PTSD in children at 1 month and at 1 year after these earthquakes (33% and 15.8%, respectively) in a non-representative sample of children has recently been published [3] and, moreover, the disaster occurred in the midst of a long European economic crisis that has well-documented negative consequences for the mental health of the general population [4].

Most of the literature on the psychopathological consequences of earthquake exposure has focused on posttraumatic stress disorder (PTSD) [5–9]. These studies suggest that its prevalence in the first 1–2 years after a disaster is variable, ranging from 5% to 60% in those directly affected to approximately 5–10% in the general population [7] and other mental disorders are also common [10,11]. For example, the prevalence of major depression after natural disasters ranges from 5.8% to 54.0% in adults in a recent meta-analytic review [12]. Different characteristics of the disaster, the area affected and methodological issues appear to explain the variability found[13–15].

One important factor influencing the risk of mental disorders after a natural disaster is a prior history of mental disorder [8,13,16–18]. Indeed, a meta-analysis has shown that the most powerful predictor of PTSD is a prior history of PTSD [19] and that a history of other psychiatric disorders is another of the most powerful risk factors in predicting PTSD [20]. Nevertheless, the great majority of studies focusing on risk factors for mental disorders after disasters have not assessed preexisting psychiatric problems [5,12,21–36]. In this study, we have therefore tried to focus on the effects of a history of PTSD and other mental disorders on post-disaster outcomes and explaining the associations of other predictors with these outcomes using data from a community epidemiological survey that was already on going in the Murcia region of Spain at the time of the Lorca earthquake [37]. A similar strategy had previously been used [16].

## Materials and methods

### Sample

The PEGASUS-Murcia (“Psychiatric Enquiry to General Population in Southeast Spain-Murcia”) project is a cross-sectional general population survey carried out between 2010 and 2012 as part of the World Mental Health (WMH) Survey Initiative (http://www.hcp.med.harvard.edu/wmh/). As described in detail previously [4,37], the project was designed to obtain regional data on prevalence, burden, treatment, and correlates of common mental disorders in a representative sample of the general adult (aged 18 or older) population of Murcia.PEGASUS-Murcia project is based on a representative sample of the adult non-institutionalized general population of the Murcia Region, one of the 17 Autonomous Communities of Spain. More details on the sampling frame, selection and weighting procedures are available elsewhere [37]. When the Lorca earthquakes occurred, the PEGASUS-Murcia project had already started but had not yet interviewed the affected area, making it possible to modify the questionnaire with the inclusion of specific questions to evaluate the effects of exposure of the population previously selected. The interviews took place between 6 and 10 months after the earthquake.

### Diagnostic assessment

A revised version of the WHO Composite International Diagnostic Interview (CIDI 3.0, hereafter referred to as CIDI), specifically adapted for use in Spain [38], was used in the PEGASUS-Murcia project. This is a comprehensive, highly-structured interview designed by the World Health Organization (WHO) to ascertain diagnoses of mental illnesses in comparative studies of the epidemiology of mental illnesses throughout the world [39]. The validity of the CIDI had been established prior to the study. In clinical reappraisal studies the CIDI has shown moderate-to-excellent for most mental disorders with the Structured Clinical Interview for DSM-IV (SCID), as the clinical gold standard [40,41]. In order to facilitate the response time, it was divided into two parts with questions in Part I administered to all respondents and those in Part II administered only to a subsample of individuals consisting of all those with Part I lifetime core disorders and a probability sub-sample of other Part I respondents. A weighting was used to adjust for the under-sampling of Part I respondents without disorders to maintain the representativeness of the Part II sample. The questionnaire pathways are described in more detail elsewhere [37]. Qualified lay interviewers carried out face-to-face interviews between June 2010 and May 2012 using CAPI (Computer Assisted Personal Interviewing). Participants from Lorca were interviewed between January and April, 2012, between nine and 11 months after the earthquake. The CIDI was modified to include questions relative to the earthquake.

Different mental disorders according to DSM-IV have been considered (i.e. mood disorders -including major depression, bipolar and dysthymia-, anxiety disorders -generalized anxiety disorder, social phobia, specific phobia, post-traumatic stress disorder, agoraphobia without panic, panic disorder, obsessive compulsive disorder and adult separation anxiety disorder-, substance disorders–alcohol and drug abuse and/or dependence- and impulsive disorder–oppositional-defiant, conduct and attention deficit disorders). As described previously [4], all diagnoses specifically excluded organic causes and without diagnostic hierarchy rules with the exception of major depressive disorder, dysthymia, general anxiety disorder and oppositional-defiant disorder. For substance use disorders, abuse was defined with or without dependence in recognition of abuse being a stage in the progression to dependence.

Prevalence estimates of mental disorders were determined by whether respondents’ symptomatology met the 12-month diagnostic criteria for a mental disorder. Lifetime prevalence of mental disorders prior to the earthquakes was determined on the basis of whether respondents had a history of mental disorder with an Age-of-onset (AOO) a year prior the interview. Retrospective AOO reports were obtained in the CIDI using a series of questions designed to avoid the implausible response patterns obtained when using the standard CIDI age-of-onset questions [42].

### Severity of 12-month disorders

Respondents were categorized as having severe mental illness [43] if they were diagnosed with 12-month bipolar I, if they attempted suicide in the last 12-months and had any 12-month diagnosis, if they had substance dependence with physiological symptoms or if they had more than one 12-month diagnosis and a high level of impairment on any of the Sheehan scales [44] including disability in work role performance, household maintenance, social life and intimate relationship.

### Socio-demographic variables

As described previously [4], socio-demographic variables included: age at interview (categorized as 18–24, 25–34, 35–49, 50–64, 65+); sex (male/female); marital status (married-cohabitating, separated-widowed-divorced, never married); completed years of education (four categories: None or primary: 0–7 years; Basic: 8–11 years; Secondary: 12–15 years and College: 16 or more years of education); and family income. The latter is the sum of all pre-tax income in the past 12 months, including salaries earned by all members of the household plus all sources of other income (e.g., government transfers, pensions and investment income). Respondents report each income component in a range of Euros (e.g., 14,000–14,999 €), which we converted to the midpoint of the range (e.g., 14,500 € in the above example) to simplify calculations. If a respondent did not report any single component of income, predictive mean matching method for imputation was used. Per capita income was then calculated for each family. This is the family income divided by the number of people in the household, as reported in the household listing. In order to compare income across countries within the WMH Surveys we created a four-category income scale. A respondent was assigned a category on this scale based on the per capita income of the respondent's family divided by the median income for Spain. The household is in the low, low-average, high-average or high categories if this ratio is 0.5 or less, over 0.5 to 1.0, over 1.0 to 2.0, or over 2.0, respectively. Employment status was based on six categories (working, student, homemaker, retired/disabled, unemployed and others).

### Earthquake-related stressors

Shortly after the earthquake, the questionnaire was modified to include a specific module to evaluate the severity of the exposure to the catastrophe. It included 22 structured questions exploring the exposure to different stressors related to the earthquake. Each one of them were categorized into 10 different categories of stressors coded as dichotomous variables (yes/no): (1) Life-threatening experience for you or for close contacts; (2) Death of family members, friends or neighbours; (3) Serious personal injury; (4) Serious injury to family members, friends or close neighbours; (5) Buried or trapped in rubble; (6) Financial loss; (7) Property (home) seriously damage or destroyed; (8) Greater family or household duties or living with relatives, friends, neighbours or strangers; (9) Neighbourhood destroyed or seriously damaged; and (10) Job affected or loss. Full text of the interview schedule that includes the complete set of stressor questions is provided in S1 Table. Two scores were then calculated to evaluate the impact of the earthquake on individuals. A Global Earthquake Stressor Score (GESS) (range: 0–10) was calculated by adding the individual score of each of the 10 categories. The second score, the Earthquake’s Experienced Stress (EES), was based on a specific question (“*On a scale between 0 and 10 where 0 means “no stress at all” and 10 means “the most stress you can imagine a person having*,*” what number describes how much stress you experienced as a result of the earthquake*?“).

### Statistical analysis

All the estimates were obtained using the sample weights to adjust for differential probabilities of selection. Weighting procedures are described in more details elsewhere [37]. Prevalence estimates are provided and expressed in weighted percentages with the standard error (se) and differences are measured by chi-squared test. Lifetime prevalence prior to the earthquake was estimated as the weighted percentage of respondents fulfilling DSM-IV criteria for a given disorder up to a year before their age at interview. Correlates of mental disorders were investigated by multiple logistic regression analyses, with PTSD, any anxiety and any mood disorder as the dependent variable. The small number of individuals with impulse or substance disorder was excluded from further analyses. Socio-demographic characteristics (sex, age, income, marital status, education and employment status) and the earthquake’s related stressors (GEES and EES), with an exposure value of 0 in those participants not exposed to the earthquake, were entered as independent variables. All analyses were weighted, and standard errors, confidence intervals and inference tests were obtained using the Taylor series linearization method [45] implemented in the STATA software Version 10.0, using the svy command with weights specified as “probability weights” to adjust for the effects of weighting and clustering on the precision of estimates [46].

All participants signed a written informed consent before interview and the Clinical Research Ethics Committee of the University Hospital Virgen de la Arrixaca of Murcia approved the protocol [37]. The database of personal information was registered with the National Data Protection Agency. The study is reported in accordance with the STROBE (Strengthening The Reporting of Observational Studies in Epidemiology) statement guidelines (S2 Table) [47].

## Results

A total number of 2,621 participants, overall response rate of 67.7%, were interviewed in the general survey and previous analyses [4], comparing sociodemographic data in terms of (sex, age and Health Care Areas) suggested that our sample was representative of the general population of the Region of Murcia. The number of participants from Lorca was 412 (18% of the total sample with a response rate of 71%). Participants from the rest of Murcia were 2,206 (82% of the total sample with a response rate ranging from 62% to 70% according to other Health Areas). Description of the socio-demographic characteristics of the participants is presented in Table 1.

**Table 1:** Sociodemographic distribution of participants from Lorca and the rest of Murcia.

	Total		Lorca		Rest of Murcia		p-value‡
	N	Weighted %	N	Weighted %	N	Weighted %	
**Participants**	**2621**		412	18	2209	82	
Sex^*^							**0.025**
Male	1192	50.5	183	52.6	1009	50.2	
Female	1429	49.5	229	47.4	1200	49.8	
Age^*^							0.394
18–24	179	8.0	29	8.3	150	8.0	
25–34	431	20.3	64	22.5	367	20	
35–49	841	31.6	124	29.2	717	31.9	
50–64	635	21.1	102	19.3	533	21.3	
65+	535	19.0	93	20.8	442	18.8	
Marital status^*^							0.614
Married/Cohabitating	1879	71.1	302	72.4	1577	71.0	
Separated/Widowed/Divorced	318	10.9	45	10.0	273	11.0	
Not Married	424	18	65	17.7	359	18.0	
Education^*^^#^							0.546
None or Primary	671	24.1	141	30.6	530	23.3	
Basic	830	31.8	119	29.4	711	32.2	
Secondary	599	24.4	77	20.5	522	25.0	
College	521	19.6	75	19.5	446	19.6	
Income^**^^†^							0.194
Low	370	25.0	33	11.8	337	26.7	
Low-Average	452	30.1	84	32.9	368	29.7	
High-Average	384	27.2	79	39.2	305	25.6	
High	253	17.8	45	16.2	208	18.0	
Employment^*^							0.720
Working	1331	52.0	199	50.8	1132	52.2	
Student	106	4.6	16	5.4	90	4.4	
Homemaker	329	11.7	55	11.3	274	11.8	
Retired/disabled	549	19.2	94	20.3	455	19.0	
Unemployed	252	10.7	37	10.0	215	10.8	
Other	54	1.9	11	2.1	43	1.8	

^**‡**^Chi-square test. Level of significance at the 0.05 level, two-sided test.

^*^ Weighted percentages calculated using part 1 weights.

^**^ Weighted percentages calculated using part 2 weights.

# Completed years of education (four categories: None or primary: 0–7 years; Basic: 8–11 years; Secondary: 12–15 years and College: 16 or more years of education).

† Family income is defined as a four-category income scale calculated as the ratio of family income in the past 12 months to the median income for Spain. Low income is defined as less than or equal to 0.5, low average as 0.5 to 1.0, high average as 1.0 to 2.0, and high as over 2.0.

### Earthquake-related stressors

Table 2 provides descriptive statistics for exposure to different earthquake related stressors both for the prevalence of each individual stressor and for the number of stressors (0–10). Almost 93% of the sample from Lorca was exposed to at least 1 individual stressor, 70% of them to more than one stressor. The EES of the participants were 2.7 stressors (95%CI = 1.6 to 3.7). More than half of participants from Lorca were exposed to the destruction or seriously damaged of their neighbourhood (69.7%) or affected their familial duties or changed the people they lived with (50.1%). A quarter had a financial loss and almost 17% suffer the death of a family member, friend of neighbour. Only 1.2% declared to have been seriously injured, although 3.6% were buried or trapped in rubble.

**Table 2:** Descriptive statistics for earthquake-related stressors in Lorca’s subsample (n = 412).

	N*	Prevalence (%)	SE
**Individual stressors**			
- Neighbourhood destroyed or seriously damaged	276	69.7	0.3
-More family or household duties or living with relatives, friends, neighbours or others	192	50.1	3.3
-Life-threatening experience for you or for close family members	176	45.5	5.1
-Property (home) seriously damage or destroyed	126	32.6	0.5
-Financial loss	103	25.0	3.3
-Job affected or loss	75	18.0	3.5
-Death of family members, friends or neighbours	69	16.8	4.7
-Seriously injured family members, friends of close neighbours	29	6.4	0.1
-Buried or trapped in rubble	15	3.6	2.2
-Serious personal injury	5	1.2	0.5
**Stressors, N°**			
0	28	7.3	2.9
1	73	17.8	3.1
2	95	24.5	1.3
3	82	20.4	6.1
≥4	117	30.1	1.4
Any (≥1)	367	92.7	2.9
**Global Earthquake’s Stressor Score (GESS)**† (N, Mean, [95% CI])	395	2.7	0.1
**Earthquake’s Experienced Stress (EES)**‡ (N, Mean, [95%CI])	369	6.4	0.3

* Percentages, means, and 95% CI calculated using part 1 weights in the sample of Lorca.

**† **GESS: Global Earthquake Stressor Score (range: 0–10)

**‡ **EES: Earthquake’s Experienced Stress (range: 0–10)

### Lifetime and 12-month prevalence of mental disorders and severity

The upper panel in Table 3 shows the distribution of lifetime prevalence of mental disorders prior to the earthquake. The area of Lorca had significantly lower lifetime prevalence of other anxiety, any impulsive/control, any substance and any mental disorders than the rest of Murcia prior to the earthquake. There were no significant differences in terms of PTSD and any mood disorder.

**Table 3:** Lifetime mental disorders prior to earthquakes and 12-month post-earthquake mental disorder prevalence in relation to prior lifetime mental disorders in Lorca versus the rest of Murcia.

	Lorca								Rest of Murcia					
					Prior lifetime mental disorder^‡^							Prior lifetime mental disorder^‡^			
	Total				Yes			No		Total		Yes		No	
	N		% (se)		N	% (se)	N	% (se)	N	% (se)	N	% (se)	N	% (se)
**I. Lifetime mental disorders prior to time of earthquake** ^**¥**^														
- PTSD	17		4.9 (1.4)			-		-	41	2.2 (0.5)		-		-
- Other anxiety	38		**8.4** **(1.2)** ^*^			-		-	247	**12.9 (1.3)**		-		-
- Any mood	50		11.9 (1.9)			**-**		**-**	308	13.6 (0.9)		**-**		-
- Any impulse/control	2		**0.4** **(0.1)** ^*^			-		-	33	**2.7 (0.6)**		-		-
- Any substance	13		**4.4** **(0.3)** ^*^			-		-	101	**8.8 (1.2)**		-		-
- Any	84		**20.7** **(1.9)** ^*^			-		-	536	**32 (2.0)**		-		-
**II. Twelve month mental disorder**														
- PTSD	15		**3.6** **(1.1)** ^*^		12	**14.5 (5.4)** ^**§**^	3	**0.8 (0.3)** ^**#**^ ^&^	11	**0.5 (0.2)**	10	**1.6 (0.5)**	1	**0.0 (0.0)** ^&^
- Other anxiety	25		**5.3** **(0.1)** ^*^		24	24.4 (3.8)	1	0.3 (0.3) ^&^	176	**9.2 (1.1)**	162	26.7 (3.0)	14	1.0 (0.3) ^&^
- Any mood	22		5.3 (2.4)		18	20.4 (2.8)	4	1.4 (1.1) ^&^	151	6.8 (0.5)	119	19.7 (2.2)	32	2.6 (0.7) ^&^
- Any impulse/control	0		0 (-)		0	-	0	-	6	0.4 (0.2)	6	-	0	-
- Any substance	1		0.2 (0.2)		1	-	0	-	16	1.1 (0.4)	16	-	0	-
- Any 12-month	54		**12.8** **(0.7)** ^*^		47	52.8 (4.9)	7	2.3 (0.9) ^&^	310	**16.8 (1.4)**	266	45.2 (3.5)	44	3.4 (0.7) ^&^
- Any serious disorder^†^	20		5.5 (0.4)		16	**20.5 (2.0)** ^**§**^	4	1.5 (0.6) ^&^	83	4.7 (0.9)	71	**13.2 (2.3)**	12	0.6 (0.3) ^&^

^‡^ Prior lifetime disorder: cases are defined as any lifetime disorder prior to time of earthquake.

^† ^Any serious disorder: cases are defined as any 12-month disorder resulting in severe mental illnesses.

^**¥**^ Lifetime disorder prior to time of earthquake: cases are defined as lifetime cases for which the difference between the age at the interview and the age at the time at which the disorder appeared was greater than one year (age in entire years).

* P-value ≤ 0.05 for the comparison of Lorca versus rest of Murcia.

& P-value ≤ 0.05 for the comparison among those with and without prior lifetime mental disorders.

§ P-value ≤ 0.05 for the comparison of Lorca versus rest of Murcia among people with some prior lifetime disorder.

# P-value ≤ 0.05 for the comparison of Lorca versus rest of Murcia among people with no prior lifetime disorder.

All the estimates in blocks I and II have been calculated using the corresponding sample weights as in [3]. In block II, part 2 weights have been used to calculate prior lifetime disorder.

The second half of Table 3 shows results for the 12-month overall prevalence stratified by prior lifetime mental disorders in the areas of Lorca and in the rest of Murcia. A similar pattern to that found for lifetime disorders is observed for the 12-month prevalence of other anxiety and any mental disorders. However, the 12-month prevalence of PTSD is almost 7 times higher in Lorca than in the rest of Murcia. The higher prevalence is observed both in those with and without a prior history of disorder. Noteworthy is the fact that the maximum difference is found within those with a previous lifetime prevalence of any mental disorder (14.5% vs 1.6%, respectively). Severe mental disorders in those from Lorca compared with the rest of Murcia is only significantly higher in participants with a lifetime prevalence of mental disorder prior to the earthquakes.

### Risk of mental disorders associated with exposure to the earthquake

Table 4 shows the results of the two different multivariate logistic regression models. Model 1 reflects the risk of 12-prevalence of each mental disorder category (PTSD, any other anxiety and any mood disorders) and any severe 12-month mental disorders adjusted for socio-demographic variables and lifetime prevalence of mental disorders prior to the earthquake (the latest only when the total sample is analyzed). Only 12-month prevalence of PTSD remains significant in the model in the general sample and among those with and without a prior history of any mental disorder.

**Table 4:** Multivariate logistic regression analyses of 12-month of PTSD, any other anxiety, mood and any severe 12-month disorder in Lorca compared to the rest of Murcia.

	Model 1^a^		Model 2^b^					
	Lorca		Lorca		GESS^c^		EES^d^	
Groups of outcomes	OR	95% CI	OR	95% CI	OR	95% CI	OR	95% CI
**I. Total sample**								
- 12-month PTSD	**13.78**	**(1.56; 121.49)**	0.04	(0.00; 14.77)	**4.06**	**(3.29; 5.01)**	1.12	(0.71; 1.76)
- 12-month any other anxiety disorder	0.67	(0.34; 1.35)	**0.13**	**(0.04; 0.46)**	**0.56**	**(0.32; 0.99)**	**1.43**	**(1.00; 2.05)**
- 12-month any mood disorder	0.71	(0.29; 1.73)	**0.09**	**(0.05; 0.15)**	**1.34**	**(1.01; 1.77)**	**1.12**	**(1.05; 1.19)**
- Any severe 12-month disorder	1.33	(0.70; 2.5)	**0.34**	**(0.16; 0.73)**	**1.59**	**(1.15; 2.19)**	0.95	(0.82; 1.11)
**II. Among people with a prior history of any mental disorder**^**e**^								
- 12-month PTSD	**13.76**	**(1.17; 161.18)**		0.89	(0.01; 115.06)	**4.46**	**(2.80; 7.11)**	0.74	(0.53; 1.03)
- 12-month any other anxiety disorder	0.94	(0.49; 1.81)		0.09	(0.00; 7.89)	0.55	(0.26; 1.15)	1.57	(0.72; 3.4)
- 12-month any mood disorder	0.75	(0.37; 1.5)		0.11	(0.01; 1.02)	1.17	(0.87; 1.56)	1.17	(0.84; 1.64)
- Any severe 12-month disorder	1.27	(0.66; 2.45)		1.10	(0.20; 6.1)	1.34	(0.91; 1.97)	0.89	(0.6; 1.32)
**III. No prior history of any mental disorder** ^**e**^								
- 12-month PTSD	**92.37**	**(7.18; 1188.65)**	7.41	(0.09; 581.17)	**1.66**	**(1.10; 2.51)**	1.15	(0.86; 1.54)
- 12-month any other anxiety disorder	0.10	(0.00; 44.08)	0.02	(0; 4.75)	**0.62**	**(0.49; 0.78)**	**1.47**	**(1.27; 1.7)**
- 12-month any mood disorder	0.35	(0.02; 7.55)	**0.06**	**(0.01; 0.37)**	**1.38**	**(1.09; 1.74)**	1.00	(0.96; 1.04)
- Any severe 12-month disorder	4.04	(0.28; 58.55)	0.90	(0.06; 13.96)	1.53	(0.96; 2.44)	0.91	(0.77; 1.09)

All the estimates have been obtained using the part 2 weights.

^**a**^ **Model 1**: Each row represents the risk (Odds ratio, OR and its 95% confidence interval, 95%CI) of being exposed to the earthquake, represented by a geographical variable (Lorca vs rest of Murcia, as the reference) calculated by a multivariate logistic regression model of the specific diagnostic categories described (yes/no) as the dependent variable. In this model, all ORs are adjusted for sociodemographic variables (sex, age, marital status, education and employment described in Table 1). In the case of the total sample, lifetime disorders prior to time of earthquakes (PTSD–yes/no- and the number of any other anxiety, mood, impulse/control and substance disorders) was also included in the model.

^**b**^ **Model 2**: includes Model 1 and additionally adjusted for GEES (Global Earthquake Stressor Score) and EES (Earthquake’s Experienced Stress).

^**c**^**GESS**: Global Earthquake Stressor Score, considered as continuous variable (range: 0–10) with zero for those from the rest of Murcia who have not been exposed to the earthquakes.

^**d**^**EES**: Earthquake’s Experienced Stress, considered as continuous variable (range: 0–10) with zero for those from the rest of Murcia who have not been exposed to the earthquakes.

^**e**^ In groups II and III, education and employment have been grouped in two different dichotomous categories (None or Primary vs Basic/Secondary/College and Working vs other employment status, respectively) in both models for the stratified approach based on prior history of any mental disorder. Specifically, for the analyses of those with no prior history of any mental disorders (group III), an interaction variable between the geographical variable (Lorca vs rest of Murcia) and prior history of any mental disorder due to the sparse number of cases was included.

Nevertheless this association with PTSD disappears when the model is also adjusted by the earthquake’s related variables, GEES and EES (Model 2). In particular, GEES seems to explain the higher prevalence of PTSD in Lorca. The other significant variables in predicting risk of 12-month of PTSD are prior lifetime PTSD and any impulse/control disorders (see S3 Table). Both GEES and EES also influence the risk of 12-month prevalence of any other anxiety disorder (in the total sample and in those with no prior history of any mental disorder) and any mood disorder in the total sample. Severity of 12-month mental disorders is modified only in the general sample by GEES. Prior lifetime prevalence of any other anxiety disorder significantly increases the risk of having any other anxiety disorder after the earthquakes. Being female, having low levels of income and a previous lifetime history of any mood disorders also increased the risk of having any mood disorder following the earthquakes.

## Discussion

This study investigated and compared the 12-month prevalence of different mental disorders in an earthquake affected area (Lorca) with a non-affected community (the rest of areas of Murcia) as a comparison group. Twelve-month prevalence of PTSD was significantly higher compared with a non-affected adjacent region. The main risk factors that explained these differences are related to the degree of exposure and a prior lifetime history of mental disorders.

Twelve-month prevalence of PTSD, any other anxiety and any mood disorder in Lorca after the earthquakes were 3.6%, 5.3% and 5.3% respectively and almost 13% of the affected population had some mental disorder after the event. Only 12-month prevalence of PTSD is higher in those exposed compared to the rest of the adjoining non-affected region. Thus, the impact of this exposure on mental health seems to be specific to PTSD.

There are several challenges in making direct comparisons between our results and those previously reported due to differences in the disaster itself, the period of time between the disaster and the PTSD assessment, the measures used, and the extent of the exposure and sampling methods [7]. Moreover, the time period between the disaster and the assessment influences the prevalence rates of PTSD due to spontaneous remission [48]. Therefore, the longer period of time produces the highest spontaneous remission rates and the lowest PTSD prevalence. In terms of measures used, the CIDI, used in our study, provides the categorical diagnosis of mental disorders based on DSM-IV, while other studies used different PTSD or depression symptom scales [5,21,24,29,34,36,49]. Prevalence varies according to the extent of exposure. For example, prevalence rates of suspected PTSD were 47.3% in heavily affected areas while in other areas the prevalence decreased to 10.4% [26]. It also varied according the selection of the population studied. For example, 43% of the participants selected from directly affected people from camps and prefabricated housing sits was estimated to have PTSD [17], while, in the same earthquake, the prevalence of PTSD comes down to 23% or 14% in two randomly selected samples [16].

Moreover, differences between countries (high income vs middle or low income countries) make comparisons more difficult as, in general, earthquakes causes less extensive devastation and casualties in developed countries [16]. Prevalence of PTSD and major depression 2–4 months following the 2010 Haiti earthquake was 24.6 and 28.3% respectively [50]. A recent study has described the prevalence of DSM-IV PTSD after natural disasters in the context of the World Mental Health Surveys [18]. Data from randomly-selected traumatic experiences related to natural and human-made disasters reflects that the prevalence of PTSD varied among high income countries from 0.1% (95%CI: 0.0–0.2) to 3.8% (0.0–11.2). In our current paper, PTSD prevalence is within the range of results as the previous published paper using the same diagnostic instrument (the CIDI), sampling methods and analyses.

There was a decrease in the prevalence of other anxiety disorders in Lorca, compared to the rest of Murcia. A considerable overlap between symptoms of PTSD and those related to other psychiatric conditions (e.g.: generalized anxiety disorders (GAD), panic and obsessive-compulsive disorders among others) has been described [51,52] and there is a considerable gap in our knowledge of longitudinal trajectories between symptoms and diagnostic categories of affective and anxiety disorders [53], specifically after a natural disaster. The use of the CIDI as a diagnostic instrument providing diagnostic categories based on DSM-IV criteria makes this possibility less plausible. It might also be possible that the rapid structured institutional response to attend casualties after the earthquakes and their potential effectiveness in the reduction of affective and anxiety symptomatology [54] might have had an asymmetric response within diagnostic categories by reducing the 12-month prevalence of other anxiety, mood and substance disorders at the time of the survey. This intervention with the high heterogeneity of remission of PTSD in adults without any specific treatment [48] might explain our results.

Secondly, the presence of a previous history of psychiatric disorder independently increased the risk of subsequent mental disorders after exposure to the earthquake, consistent with previous studies [7,8,16–18,20]. Moreover, the increased risk seemed to be diagnostic-specific in the multivariate model in our study (see S2 Table): prior PTSD and any impulsive/control disorder remained as risk factor for 12-month PTSD prevalence, any other prior anxiety disorder was the only prior mental disorder for 12-month other anxiety disorders and only any prior mood disorders for 12.month any mood disorder. This specificity has also been described specifically for PTSD, where prior lifetime PTSD was the most powerful predictor for PTSD among other prior lifetime mental disorders [19]. Further research is needed to investigate the relative importance of prior mental disorders as pre-traumatic risk factors.

Thirdly, the intensity of the earthquake exposure, measured as the number of stressors (GESS) and the emotional impact (EES), is an important variable explaining differences of 12-month PTSD prevalence and severity between Lorca and rest of Murcia and these results are consistent with the published literature in two meta-analyses [12,20]. Finally, being female (for other anxiety and mood disorders) and low or low-average self-reported income (for any mood disorder) remained significant risk factors in the final multivariate model. Female sex and income level have been consistently reported to be associated with mental problems after a natural disaster [7,12,20]. Women have been described to be more sensitive to threats and to interpret disasters more negatively than men [55], but contradictory results in studies looking for gender effects have also been described [8]. People with a lower income level might be more affected, especially in the context of an economic crisis [4]. The lack of significance of other socio-demographic correlates (such as education, marital status and age) should be interpreted with caution and it is unclear whether this situation is related to Lorca’s specific disaster context or to methodological issues [7]. The inclusion of potential mediators in the multivariate analyses might obscure their relationship with mental disorders in this particular study, as lower educational level, not married and younger age have been previously described, though with some inconsistencies [7,8,12,20].

The strengths of the study are the selection of the population previous to the earthquakes, the inclusion of a wide range of mental disorders, and the inclusion of two samples, one exposed (from Lorca) and the other non-exposed (from the rest of Murcia) using the same sampling method and diagnostic instrument. Nevertheless, our results should be interpreted within the context of some limitations. First, the overall response rate of 67.7% is not completely satisfactory but is above the 60% conventionally considered as a minimum standard in general population surveys [56]. Participants from Lorca reached a 70% of response rate. Participants interviewed in the general survey were comparable from available census data from Murcia suggesting a representativeness of the general population [4]. Second, diagnoses were based on a fully structured lay-administered interview rather than semi-structured clinical interviews. This limitation in reporting DSM-IV diagnoses has been evaluated with a moderate-to-excellent concordance as regards the diagnosis of most mental disorders [40,41] and it is currently widely used in epidemiologic surveys of general population all over the world [39]. Third, the self-report assessment may reduce the reliability of some information that might have underestimated the risk, especially that related to lifetime mental disorders prior to the earthquakes. Fourth, the specific module added to the CIDI to measure the earthquake-exposure was not implemented in the non-affected areas (the rest of Murcia) and some areas were interviewed before earthquakes. These might have underestimated the PTSD prevalence due to indirect earthquakes’ exposure, such as television coverage [57]. Finally, the cross-sectional design limits the interpretation of the associations found being interpreted as causal.

Research on post-disaster psychological consequences is complicated because of the specific difficulties inherent to the complex logistic organization needed to start a survey in a context of the post-disaster circumstances after an unpredicted disaster [58] and the high heterogeneity found in published literature related to: i) the disaster, such as the type of trauma or duration of the exposure; ii) the characteristics of the population exposed, including the region/country affected; and iii) different methodological issues, such as study design, sample and diagnostic instruments selected [13–15]. Whenever a catastrophe occurs, it constitutes a unique opportunity to add new evidences to improve our knowledge of mental health epidemiologic research on natural disasters, and to provide information for service managers, professionals responsible for prevention-treatment interventions and interested researchers [5,20,59,60]. Psychological interventions to prevent mental disorders after the exposure to earthquakes should be focused on people at higher risk and our study shows that this should particularly include those with a higher direct exposure to the natural disaster and those with a prior history of mental disorders.

## Conclusions

In summary, the main outcome of our study is that PTSD and depression feature prominently following an earthquake and our study shows that the likelihood of their occurrence may be predicted both by a history of prior mental disorders and the level of exposure, this latter being one of the most important risk factors for the development of a subsequent mental disorder. This identification should be of assistance to healthcare planners who should thus focus their attention and resources on this particularly vulnerable group.

## Supporting information

S1 Table Earthquake´s exposure section. (DOC)

S2 Table STROBE (Strengthening The Reporting of Observational Studies in Epidemiology) checklist of items that should be included in reports of *cross-sectional studies*. (DOCX)

S3 Table Twelve-month prevalence of PTSD, any anxiety disorder and mood disorders according to socio-demographic and earthquake’s exposure variables. **ᶧ** p-valor < 0.05; ^&^Adjusted odds ratio (OR) and 95% confidence interval (95% CI): all the variables in the Table are included in the model. The variables GESS and EES are zero for the rest of Murcia. These variables have been considered as continuous variables. All the estimates have been obtained using the part 2 weights. **‡** Prior lifetime mental disorders to earthquakes. **†** PTSD: Post-traumatic Stress Disorder.
(DOC)
