# Supplementary material for: Post-Traumatic Stress Disorder and other mental disorders in the general population after Lorca’s earthquakes, 2011 (Murcia, Spain): A cross-sectional study
Source: PLoS One. 2017 Jul 19;12(7):e0179690. doi: 10.1371/journal.pone.0179690 (PMC5516965; doi:10.1371/journal.pone.0179690)
Supplement: S1 Table — (DOC) [file pone.0179690.s001.doc]

**Supporting Information:**

**S1 Table: Earthquake´s exposure section**

The World Mental Health Composite International Diagnostic Interview (CIDI 3.0) is described at <https://www.hcp.med.harvard.edu/wmhcidi/>. All sections can be viewed and/or downloaded at <https://www.hcp.med.harvard.edu/wmhcidi/instruments_capi.php>, except the following, related to the earthquake’s exposure. The Spanish version of the whole CIDI is only available on request to the editorial committee.

**SECCIÓN DEL TERREMOTO (EARTHQUAKE´S SECTION)**

PT7. Las siguientes preguntas están relacionadas con el terremoto que ocurrió en Lorca el pasado 11 de mayo de 2011. En una escala entre 0 y 10, donde 0 significa “sin ningún tipo de ansiedad y/o estrés” y 10 significa “el mayor estrés que usted cree que una persona pueda tener”, qué número describiría la cantidad de estrés que usted experimentó como resultado del terremoto?

The next questions are about the earthquake that occurred in Lorca, Spain on May 11, 2011. On a scale between 0 and 10 where 0 means “no stress at all” and 10 means “the most stress you can imagine a person having,” what number describes how much stress you experienced as a result of the earthquake?

Número NUMBER

NO LO SABE DON’T KNOW 98

REHUSA REFUSED 99

PT8. INTERVIEWER CHECKPOINT: (See **PT7**)

**PT7** CODED ‘0’, ‘1’, ‘2’ OR ‘3’…………1 GO TO NEXT SECTION

ALL OTHERS 2

PT9. ¿Cual fue la situación más estresante que le pasó como resultado del terremoto? (TRANSCRIBIR LAS PALABRAS TEXTUALES)

What was the most stressful thing that happened to you as a result of the earthquake? (RECORD VERBATIM)

NO LO SABE DON’T KNOW 98

REHUSA REFUSED 99

**_____________________________________________________________________________**

**_____________________________________________________________________________**

**_____________________________________________________________________________**

| PT1.  Las siguientes preguntas están relacionadas con su experiencia en el terremoto. ¿Cuál de las siguientes situaciones le sucedieron como resultado del terremoto?:  The next questions are about your experiences in the earthquake. Which of the following things happened to you as a result of the earthquake: |  | | | |
| --- | --- | --- | --- | --- |
|  | SI **(1)** | **NO**  **(5)** | **DK**  **(8)** | RF **(9)** |
| PT1a. ¿Su casa sufrió grandes daños? Your home was seriously damaged? | 1 | 5 | 8 | 9 |
| PT1b. ¿Su casa fue destruida? Your home was destroyed? | 1 | 5 | 8 | 9 |
| PT1c. ¿Se vió obligado a vivir con familiares con los que no vivía antes del terremoto?, You had to live with relatives who did not live with you before the earthquake? | 1 | 5 | 8 | 9 |
| PT1d. ¿Se ha visto obligado a vivir con amigos o vecinos?. You had to live with friends or neighbors? | 1 | 5 | 8 | 9 |
| PT1e. ¿Tuvo que vivir con extraños o gente que no conocía bien?. You had to live with strangers or with people who you did not know very well? | 1 | 5 | 8 | 9 |
| PT1f. ¿El lugar donde usted trabajaba ha sufrido daños graves o ha sido destruido?. The place where you worked was seriously damaged or destroyed? | 1 | 5 | 8 | 9 |
| PT1g. ¿Perdió su trabajo?, You lost your job? | 1 | 5 | 8 | 9 |
| PT1h. ¿Ha tenido que reducir la cantidad de tiempo que trabajaba?. You had to reduce the amount of time you worked? | 1 | 5 | 8 | 9 |
| PT1i. ¿Ha tenido que asumir más obligaciones laborales?. You had to take up more work duties? | 1 | 5 | 8 | 9 |
| PT1j. ¿Tuvo que asumir más responsabilidades familiares o tareas domésticas?. You had to take up more family or household duties? | 1 | 5 | 8 | 9 |
| PT1k. ¿Tuvo pérdidas económicas importantes?. You experienced serious financial loss? | 1 | 5 | 8 | 9 |
| PT1m. ¿Ha tenido que pedir un préstamo al banco/familiares/amigos?. You had to loan from bank/relatives/friends？ | 1 | 5 | 8 | 9 |
| PT1n. Durante el terremoto, ¿tuvo miedo de morir o de que personas cercanas a usted pudieran morir?  During the earthquake, were you afraid that you might die or that people close to you might die? | 1 | 5 | 8 | 9 |

PT2. ¿Cuántos de sus familiares cercanos murieron en el terremoto? How many of your close family members died in the earthquake?

NUMBER

NO LO SABE DON’T KNOW 98

REHUSA REFUSED 99

PT3. ¿Cuántos de sus amigos o vecinos cercanos murieron en el terremoto? How many of your friends or close neighbors died in the earthquake?

NUMBER

NO LO SABE DON’T KNOW 98

REHUSA REFUSED 99

PT4. ¿Cuántos de sus familiares cercanos fueron heridos gravemente en el terremoto? How many of your close family members were seriously injured in the earthquake?

NUMBER

NO LO SABE DON’T KNOW 98

REHUSA REFUSED 99

PT5. ¿Cuántos de sus amigos o vecinos cercanos fueron heridos gravemente durante el terremoto? How many of your friends or close neighbors were seriously injured in the earthquake?

NUMBER

NO LO SABE DON’T KNOW 98

REHUSA REFUSED 99

PT5a1. ¿Tenía un seguro para su vivienda en el momento del terremoto? Did you have property insurance at the time of the earthquake?

SI YES 1

NO 5

NO LO SABE DON’T KNOW 8

REHUSA REFUSED 9

PT5b. ¿Estaba usted solo o con alguien más en el momento del terremoto? Were you alone or with someone else at the time of the earthquake?

SOLO ALONE 1

CON ALGUIEN MÁS WITH SOMEONE ELSE 5

NO LO SABE DON’T KNOW 8

REHUSA REFUSED 9

PT5c. ¿Quedó usted enterrado o atrapado entre los escombros? Were you buried or trapped in rubble?

SÍ YES 1

NO NO 5 **GO TO PT5e**

NO LO SABE DON’T KNOW 8 **GO TO PT5e**

REHUSA REFUSED 9 **GO TO PT5e**

PT5d. ¿Cuánto tiempo estuvo usted enterrado o atrapado entre los escombros? How long were you trapped in rubble?

NÚMERO DE DÍAS NUMBER OF DAYS

NO LO SABE DON’T KNOW 98

REHUSA REFUSED 99

**GO TO PT5g**

PT5e. ¿Estuvo usted sin poder irse en el lugar del desastre tras el terremoto? Were you trapped in the disaster site after the earthquake?

SI YES 1

NO NO 5 **GO TO PT5g**

NO LO SABE DON’T KNOW 8 **GO TO PT5**g

REHUSA REFUSED 9 **GO TO PT5g**

PT5f. ¿Cuánto tiempo estuvo usted sin poder irse en el lugar del desastre tras el terremoto? How long were you trapped in the disaster site after the earthquake?

NÚMERO DE DÍAS NUMBER OF DAYS

NO LO SABE DON’T KNOW 98

REHUSA REFUSED 99

PT5g. ¿Con qué gravedad fue usted herido físicamente durante el terremoto – muy gravemente, algo, no mucho, o nada? How seriously were you physically injured during the earthquake – very seriously, somewhat, not very, or not at all?

MUCHO VERY 1

ALGO SOMEWHAT 2

POCO NOT VERY 3 **GO TO PT6**

NADA NOT AT ALL 4 **GO TO PT6**

NO LO SABE DON’T KNOW 8 **GO TO PT6**

REHUSA REFUSED 9 **GO TO PT6**

PT5g.1. ¿Se ha recuperado de estas heridas – completamente, la mayor parte, algo, nada? Have you recovered from these injuries - completely, mostly, somewhat, or not at all?

COMPLETAMENTE COMPLETELY 1 **GO TO PT6**

LA MAYOR PARTE MOSTLY 2

ALGO SOMEWHAT 3

NADA NOT AT ALL 4

DISCAPACIDAD PERMANTE QUE NO SE RECUPERARÁ NUNCA (IF VOL) PERMANENT DISABILITY, WILL NEVER RECOVER 5 **GO TO PT6**

NO LO SABE DON’T KNOW 8

REHUSA REFUSED 9

PT5g.2. ¿Tiene alguna alteración permanente que nunca desaparecerá que fuera causada por el terremoto, como la pérdida de un miembro o problemas de visión o desfiguración? Do you have any permanent condition that will never go away that was caused by the earthquake, like loss of a limb or vision problems or disfigurement?

SI YES 1

NO NO 5 G**O TO PT6**

NO LO SABE DON’T KNOW 8 **GO TO PT6**

REHUSA REFUSED 9 **GO TO PT6**

PT5g.3. ¿Cómo puntuaría usted la gravedad de esta alteración en una escala de 0 a 10 donde 0 significa “nada grave” y 10 significa “extremadamente grave”?. How serious would you rate this condition on a 0-to-10 scale where 0 means “not at all” serious and 10 means “extremely serious”?

NUMBER

NO LO SABE DON’T KNOW 98

REHUSA REFUSED 99

PT5g.4. Brevemente, ¿cual es esa alteración? (RESPUESTA ABIERTA) Briefly, what is the condition? (OPEN-ENDED RESPONSE)

**_____________________________________________________________________________**

**_____________________________________________________________________________**

**_____________________________________________________________________________**

NO LO SABE DON’T KNOW 98

REHUSA REFUSED 99

PT6. ¿Cuántas de las casas de su barrio fueron destruidas o dañadas gravemente durante el terremoto – todas, la mayoría, algunas, o ninguna de ellas? How many of the homes in your (neighborhood/village) were either destroyed or seriously damaged during the earthquake – all, most, some, or none of them?

TODAS ALL 1

LA MAYORÍA MOST 2

ALGUNAS SOME 3

NINGUNA NONE 4

NO LO SABE DON’T KNOW 8

REHUSA REFUSED 9

*PT9a. INTERVIEWER CHECKPOINT.

Rs RANDOM OR WORST EVENT IN THE PTSD SECTION WAS
“NATURAL DISASTER” AND WAS THE RECENT EARTHQUAKE 1 **GO TO NEXT SECTION**

ALL OTHERS 2 **GO TO PT10**

| PT10. (RB, P X) After highly stressful experiences like the one(s) you just described, people often have problems like upsetting memories or dreams, feeling emotionally distant or depressed, and feeling jumpy or easily startled. Think of the one month after the earthquake when you had the largest number of these problems. During that month, how often did you …  (IF NEC: all or almost all of the time, most, some, a little, or none of the time?) | Tras una experiencia muy estresante como la que acaba de describir, a veces la gente tiene problemas, como por ejemplo recuerdos o sueños inquietantes, dificultades para dormirse o concentrarse, sentirse emocionalmente distante de los demás, o sentirse nervioso o sobresaltado con facilidad. Piense en el mes posterior al terremoto cuando usted tuvo el mayor número de estos problemas. Durante ese mes, ¿con qué frecuencia …  (IF NEC: todo el tiempo, casi todo el tiempo, la mayor parte del tiempo, algunas veces, pocas veces o en ningún momento?) | **TODOS O CASI TODOS**  **ALL OR ALMOST ALL**  **(1)** | **LA MAYORÍA**  **MOST**  **(2)** | **ALGO**  **SOME**  **(3)** | **UN POCO**  **A LITTLE**  **(4)** | **NUNCA**  **NONE**  **(5)** | **NO LO SABE**  **DK**  **(8)** | **REHUSA**  **RF**  **(9)** |
| --- | --- | --- | --- | --- | --- | --- | --- | --- |
| PT10a. …have repeated, disturbing memories, thoughts, or images of the earthquake – that is, you kept remembering it even when you didn’t want to – all or almost all the time, most of the time, some of the time, a little of the time, or none of the time? | … tuvo recuerdos, pensamientos o imagines inquietantes y repetidas del terremoto – es decir, usted seguía recordándolo incluso cuando usted no quería - todo el tiempo, casi todo el tiempo, la mayor parte del tiempo, algunas veces, pocas veces o en ningún momento? | 1 | 2 | 3 | 4 | 5 | 8 | 9 |
| PT10b. When you were reminded of what happened, how often did you have physical reactions like sweating, your heart racing, trouble breathing or feeling shaky? | Cuando usted recordó lo que pasó, ¿con qué frecuencia tuvo síntomas físicos como sudor, taquicardia o pulso acelerado, problemas para respirar o sentimientos de debilidad? | 1 | 2 | 3 | 4 | 5 | 8 | 9 |
| PT10c. (How often did you) avoid thinking about or talking about what happened or avoided having feelings about it? | ¿(Con qué frecuencia usted) evitó pensar o hablar sobre lo que sucedió o evitó tener sentimientos relacionados con el tema? | 1 | 2 | 3 | 4 | 5 | 8 | 9 |
| PT10d. (How often did you) avoid activities or situations because they reminded you of what happened? | ¿(Con qué frecuencia usted) evitó actividades o situaciones porque le recordaban lo que le sucedió? | 1 | 2 | 3 | 4 | 5 | 8 | 9 |
| PT10e. (How often did you) have more trouble than usual concentrating or keeping your mind on what you were doing? | ¿(Con qué frecuencia usted) tuvo más problemas de lo habitual para concentrarse o para mantener su atención en lo que estaba haciendo? | 1 | 2 | 3 | 4 | 5 | 8 | 9 |
| PT10f. How often were you more jumpy or easily startled by ordinary noises? | ¿Con qué frecuencia estaba más asustadizo o se sobresaltó por ruidos normales? | 1 | 2 | 3 | 4 | 5 | 8 | 9 |
| PT10g. How often did your thoughts or memories about any of these events interfere with the quality of your life? | ¿Con qué frecuencia sus pensamientos o recuerdos acerca de cualquiera de estos hechos interfirieron con su calidad de vida? | 1 | 2 | 3 | 4 | 5 | 8 | 9 |
| PT10h. How often did these reactions interfere with your work or personal life? | ¿Con qué frecuencia esas reacciones suyas interfirieron con su trabajo o con su vida personal? | 1 | 2 | 3 | 4 | 5 | 8 | 9 |

PT11. INTERVIEWER CHECKPOINT: SEE **PT10a-h**

3 OR MORE RESPONSES IN **PT10a-h** CODED ‘1’, ‘2’, OR ‘3’ 1

ALL OTHERS 2 **GO TO *PT15**

PT12. ¿Cuánto tiempo tras el terremoto empezó a tener los problemas que acabamos de revisar? How soon after the earthquake did you start having the problems we just reviewed?

CODE “IMMEDIATELY” OR “SAME DAY” AS “0 DAYS”

NUMERO DESDE EL INICIO ONSET NUMBER

CIRCLE UNIT OF TIME: DIAS DAYS 1 SEMANAS WEEKS 2 MESES MONTHS 3 AÑOS YEARS 4

NO LO SABE DON’T KNOW 98

REHUSA REFUSED 99

PT13. ¿Con qué frecuencia tuvo estos problemas en los últimos 30 días – todo o casi todo el tiempo, la mayor parte del tiempo, algunas veces, pocas veces y nunca?. How often did you have these problems in the past 30 days – all or almost all the time, most of the time, some of the time, a little of the time , or none of the time?

TODOS LOS DÍAS ALL 1

LA MAYORÍA MOST 2

ALGUNAS VECES SOME 3

POCAS VECES A LITTLE 4

NUNCA NONE 5

NO LO SABE DON’T KNOW 8

REHUSA REFUSED 9

PT14. INTERVIEWER CHECKPOINT: SEE **PT13**

**PT13** CODED ‘1’, ‘2’, OR ‘3’ 1 **GO TO *PT16**

ALL OTHERS 2

PT15. ¿Cuándo fue la última vez que tuvo estos problemas al menos algunas veces durante un mes entero? When was the last time you had these problems at least some of the time for a full month?

MES MONTH/___________AÑO YEAR

NO LO SABE DON’T KNOW 8

REHUSA REFUSED 9

PT15.1 INTERVIEWER CHECKPOINT

R WAS IN PART II 1 **GO TO NEXT SECTION**

R WAS NOT IN PART II 2

PT16. ¿Tuvo alguna vez un momento en su vida antes del terremoto en el que usted tuviera problemas como los descritos en la página XX de su libro de respuestas? Did you ever have a time in your life before the time of the earthquake when you had problems like those on page XX of your respondent booklet?

SI YES 1 **GO TO PT17**

NO NO 5

NO LO SABE DON’T KNOW 8

REHUSA REFUSED 9

PT16a. INTERVIEWER CHECKPOINT: SEE **PT11**

**PT11** CODED ‘1’ 1 **GO TO PT22**

ALL OTHERS 2 **GO TO NEXT SECTION**

| PT17. Think of the one month in your life before the time of the earthquake when you had the largest number of these problems. If no one month comes to mind then think of a typical bad month like that. During that month, how often did you have each of the following reactions?  (IF NEC: almost all, most, some, a little, or none of the time?) | Piense en un período de un mes antes del terremoto en el que usted tuvo el mayor número de estos problemas. Si no recuerda ningún mes, entonces piense en un típico mes malo como ese. Durante este mes, ¿con qué frecuencia tuvo cada una de las siguientes reacciones?  (IF NEC: todo el tiempo, casi todo el tiempo, la mayor parte del tiempo, algunas veces, pocas veces o en ningún momento?) | **TODOS O CASI TODOS**  **ALL OR ALMOST ALL**  **(1)** | **LA MAYORÍA**  **MOST**  **(2)** | **ALGO**  **SOME**  **(3)** | **UN POCO**  **A LITTLE**  **(4)** | **NUNCA**  **NONE**  **(5)** | **NO LO SABE**  **DK**  **(8)** | **REHUSA**  **RF**  **(9)** |
| --- | --- | --- | --- | --- | --- | --- | --- | --- |
| PT17a. How often did you have repeated, disturbing memories, thoughts, or images of earthquake stressful event – that is, you kept remembering it even when you didn’t want to – all or almost all the time, most of the time, some of the time, a little of the time, or none of the time? | ¿Con qué frecuencia usted tuvo recuerdos, pensamientos o imagines inquietantes y repetidas del terremoto – es decir, usted seguía recordándolo incluso cuando usted no quería - todo el tiempo, casi todo el tiempo, la mayor parte del tiempo, algunas veces, pocas veces o en ningún momento? | 1 | 2 | 3 | 4 | 5 | 8 | 9 |
| PT17b. When you were reminded of what happened, how often did you have physical reactions like sweating, your heart racing, trouble breathing or feeling shaky? | Cuando usted recordó lo que pasó, ¿con qué frecuencia tuvo síntomas físicos como sudor, taquicardia o pulso acelerado, problemas para respirar o sentimientos de debilidad? | 1 | 2 | 3 | 4 | 5 | 8 | 9 |
| PT17c. (How often did you) avoid thinking about or talking about what happened or avoided having feelings about it? | ¿(Con qué frecuencia usted) evitó pensar o hablar sobre lo que sucedió o evitó tener sentimientos relacionados con el tema? | 1 | 2 | 3 | 4 | 5 | 8 | 9 |
| PT17d. (How often did you) avoid activities or situations because they reminded you of what happened? | ¿(Con qué frecuencia usted) evitó actividades o situaciones porque le recordaban lo que le sucedió? | 1 | 2 | 3 | 4 | 5 | 8 | 9 |
| PT17e. (How often did you) have more trouble than usual concentrating or keeping your mind on what you were doing? | ¿(Con qué frecuencia usted) tuvo más problemas de lo habitual para concentrarse o para mantener su atención en lo que estaba haciendo? | 1 | 2 | 3 | 4 | 5 | 8 | 9 |
| PT17f. How often were you more jumpy or easily startled by ordinary noises? | ¿Con qué frecuencia estaba más asustadizo o se sobresaltó por ruidos normales? | 1 | 2 | 3 | 4 | 5 | 8 | 9 |
| PT17g. How often did your thoughts or memories about any of these events interfere with the quality of your life? | ¿Con qué frecuencia sus pensamientos o recuerdos acerca de cualquiera de estos hechos interfirieron con su calidad de vida? | 1 | 2 | 3 | 4 | 5 | 8 | 9 |
| PT17h. How often did these reactions interfere with your work or personal life? | ¿Con qué frecuencia esas reacciones suyas interfirieron con su trabajo o con su vida personal? | 1 | 2 | 3 | 4 | 5 | 8 | 9 |

PT18. INTERVIEWER CHECKPOINT: SEE **PT17a-f**

AT LEAST 2 RESPONSES IN **PT17a-h** CODED ‘1’, ‘2’, OR ‘3’ 1

**PT11** CODED ‘1’ 2 **GO TO PT22**

ALL OTHERS 2 **GO TO NEXT SECTION**

PT19. Aproximadamente, ¿qué edad tenía usted la primera vez en su vida que tuvo problemas como los que acabamos de revisar durante un período de un mes o más?

SI “TODA MI VIDA” O “TANTO COMO PUEDO RECORDAR”

PRUEBE: ¿Fue antes de que empezará el colegio?

SI NO CONTESTA SÍ, PRUEBE: ¿Fue antes de ser un adolescente?

__________ AÑOS DE EDAD

ANTES DE EMPEZAR EL COLEGIO 4

ANTES DE LA ADOLESCENCIA 12

NO ANTES DE LA ADOLESCENCIA 13
TODA MI VIDA O NO LO SÉ 998

REHUSA 999

About how old were you the first time in your life you had problems like the ones we just reviewed for one month or longer?

IF “ALL MY LIFE” OR “AS LONG AS I CAN REMEMBER,”

PROBE: Was it before you first started school?

IF NOT YES, PROBE: Was it before you were a teenager?

__________ YEARS OLD

BEFORE STARTED SCHOOL 4

BEFORE TEENAGER 12

NOT BEFORE TEENAGER 13
WHOLE LIFE OR DON’T KNOW 998

REFUSED 999

PT20. Aproximadamente, ¿cuantos años diferentes en su vida ha tenido problemas como estos por un mes o más?

NÚMERO DE AÑOS

NO LO SABE 98

REHUSA 99

About how many different years in your life did you have problems like these for one month or longer?

NUMBER OF YEARS

DON’T KNOW 98

REFUSED 99

PT20a. ¿Cuántos años tenía la última vez que tuvo problemas como los que acabamos de revisar durante un mes o más?

____________ AÑOS DE EDAD

NO LO SABE 98

REHUSA 99

How old were you the last time you had problems like the ones we just reviewed for one month or longer?

____________ YEARS OLD

DON’T KNOW 98

REFUSED 99

PT21. "¿Alguna vez en su vida ha consultado un médico, psicólogo u otro profesional debido a sus reacciones por lo ocurrido en el terremoto? (Por otro profesional nos referimos a sacerdotes, yerberos, naturistas, homeópatas, acupunturistas, curanderos, espiritistas y otros profesionales relacionados con la salud.)

Did you ever in your life talk to a medical doctor or other professional about your reactions to what happened? (By other professional we mean psychologists, counselors, spiritual advisors, herbalists, acupuncturists, and other healing professionals.)

YES 1

NO 5 **GO TO *PT22**

DON’T KNOW 8 **GO TO *PT22**

REFUSED 9 **GO TO *PT22**

*PT21a. ¿Qué edad tenía la primera vez (que usted habló con un profesional acerca de sus reacciones)?

How old were you the first time (you talked to a professional about your reactions)?

____________YEARS OLD

NO LO SABE DON’T KNOW 98

REHUSA REFUSED 99

PT22. Durante los meses anteriores a esta entrevista ¿ha recibido algún tipo de tratamiento profesional para sus reacciones debido al terremoto?

Did you receive professional treatment for your reactions to what happened at any time in the past 12 months?

SI YES 1

NO NO 5

NO LO SABE DON’T KNOW 8

REHUSA REFUSED 9

**END SECTION**
