# Supplementary material for: Post-Traumatic Stress Disorder and other mental disorders in the general population after Lorca’s earthquakes, 2011 (Murcia, Spain): A cross-sectional study
Source: PLoS One. 2017 Jul 19;12(7):e0179690. doi: 10.1371/journal.pone.0179690 (PMC5516965; doi:10.1371/journal.pone.0179690)
Supplement: S3 Table — ᶧ p-valor < 0.05; &Adjusted odds ratio (OR) and 95% confidence interval (95% CI): all the variables in the Table are included in the model. The variables GESS and EES are zero for the rest of Murcia. These variables have been considered as continuous variables. All the estimates have been obtained using the part 2 weights. ‡ Prior lifetime mental disorders to earthquakes. † PTSD: Post-traumatic Stress Disorder. (DOC) [file pone.0179690.s003.doc]

**S3 Table. Twelve-month prevalence of PTSD, any anxiety disorder and mood disorders according to socio-demographic and earthquake’s exposure variables**

|  |  | **PTSD †** | | | |  | **Any OTHER Anxiety Disorder** | | | |  | **Any Mood Disorder** | | | |
| --- | --- | --- | --- | --- | --- | --- | --- | --- | --- | --- | --- | --- | --- | --- | --- |
| **Variable** | **Label** | **OR** | **95% CI** | **Adj.**  **OR ^&^** | **95% CI ^&^** |  | **OR** | **95% CI** | **Adj.**  **OR  ^&^** | **95% CI ^&^** |  | **OR** | **95% CI** | **Adj.**  **OR  ^&^** | **95% CI ^&^** |
| **Sex** | Male | 1 | - | 1 | - |  | 1 | - | 1 | - |  | 1 | - | 1 | - |
|  | Female | 2,00 | (0.74;5.36) | 2,15 | (0.41;11.24) |  | **4,47** | **(2.56;7.81)** ᶧ | 2,01 | (0.9;4.45) |  | **3,00** | **(1.89;4.77)** ᶧ | **2,47** | **(1.39;4.39) ᶧ** |
| **Age** | 18-34 | 0,98 | (0.35;2.78) | 0,15 | (0;418.66) |  | 1,25 | (0.61;2.54) | 0,28 | (0.13;0.59) |  | 2,44 | (0.78;7.65) | 2,89 | (0.72;11.61) |
|  | 35-49 | 0,95 | (0.25;3.62) | 0,18 | (0.02;2.14) |  | 0,94 | (0.56;1.57) | 0,20 | (0.06;0.64) |  | 1,65 | (0.77;3.54) | 1,66 | (0.44;6.21) |
|  | 50-64 | 2,15 | (0.47;9.8) | 2,89 | (0.48;17.47) |  | 1,08 | (0.68;1.72) | 0,36 | (0.21;0.62) |  | 1,74 | (0.71;4.26) | 1,78 | (0.45;7.06) |
|  | > 65 | 1 | - | 1 | - |  | 1 | - | 1 | - |  | 1 | - | 1 | - |
| **Income** | Low | 1,03 | (0.22;4.84) | 1,04 | (0.33;3.23) |  | 1,69 | (0.96;2.96) | 1,46 | (0.48;4.48) |  | **2,45** | **(1.62;3.7)** ᶧ | **2,60** | **(1.35;5.00) ᶧ** |
|  | Low-Average | 1,22 | (0.17;8.7) | 0,31 | (0.03;3.69) |  | 1,36 | (0.77;2.39) | 1,63 | (0.75;3.54) |  | **2,36** | **(1.08;5.17)** ᶧ | **2,97** | **(1.08;8.18) ᶧ** |
|  | High-Average | 1,97 | (0.42;9.32) | 0,80 | (0.14;4.43) |  | 1,15 | (0.66;2.01) | 1,02 | (0.53;1.96) |  | **1,70** | **(1.04;2.79) ᶧ** | 1,68 | (0.72;3.93) |
|  | High | 1 | - | 1 | - |  | 1 | - | 1 | - |  | 1 | - | 1 | - |
| **Marital Status** | Married/Cohabiting | 1 | - | 1 | - |  | 1 | - | 1 | - |  | 1 | - | 1 | - |
|  | Sep./Widowed/Divorced | 0,81 | (0.46;1.42) | 0,74 | (0.11;4.89) |  | 1,29 | (0.62;2.71) | 0,52 | (0.28;0.96) |  | 1,88 | (0.89;3.98) | 1,17 | (0.62;2.23) |
|  | Never Married | 0,15 | (0.02;1.03) | 0,25 | (0.02;2.97) |  | 1,13 | (0.7;1.83) | 0,69 | (0.27;1.80) |  | **2,14** | **(1.08;4.25)** ᶧ | 1,96 | (0.57;6.73) |
| **Education** | None or Primary | 7,10 | (0.57;88.04) | 3,07 | (0.09;99.61) |  | 1,09 | (0.53;2.26) | 1,21 | (0.29;5.10) |  | 0,76 | (0.28;2.06) | 0,67 | (0.27;1.64) |
|  | Basic | 11,24 | (0.71;178.92) | 9,96 | (0.2;486.29) |  | 0,82 | (0.39;1.75) | 1,20 | (0.29;4.92) |  | 0,98 | (0.45;2.12) | 0,77 | (0.36;1.63) |
|  | Secondary | 0,74 | (0.01;51.16) | 0,59 | (0;881.26) |  | 0,82 | (0.52;1.3) | 0,95 | (0.39;2.31) |  | 0,65 | (0.22;1.92) | 0,63 | (0.27;1.48) |
|  | College | 1 | - | 1 | - |  | 1 | - | 1 | - |  | 1 | - | 1 | - |
| **Employment** | Working | 1 | - | 1 | - |  | 1 | - | 1 | - |  | 1 | - | 1 | - |
|  | Student | 0,78 | (0.05;11.4) | 752,54 | (0.04;1.4E+07) |  | 1,65 | (0.62;4.37) | 1,81 | (0.06;59.03) |  | 1,87 | (0.69;5.13) | 0,97 | (0.42;2.22) |
|  | Homemaker | **20,38** | **(4.5;92.38)** ᶧ | 39,33 | (0.87;1771.44) |  | **2,39** | **(1.29;4.43)** ᶧ | 0,62 | (0.19;1.98) |  | 1,11 | (0.69;1.77) | 0,75 | (0.43;1.31) |
|  | Retired/Disabled | **11,35** | **(4.28;30.13)** ᶧ | 39,91 | (0.53;3013.5) |  | 0,75 | (0.33;1.7) | 0,26 | (0.10;0.70) |  | 0,85 | (0.37;1.96) | 1,96 | (0.54;7.04) |
|  | Unemployed | **30,53** | **(8.73;106.76)** ᶧ | 43,07 | (0.99;1864.01) |  | 0,74 | (0.44;1.25) | 1,51 | (0.47;4.87) |  | **2,23** | **(1.05;4.75)** ᶧ | 1,60 | (0.73;3.51) |
|  | Others | **59,18** | **(7.62;459.55)** ᶧ | 15,56 | (0.5;481.76) |  | 1,65 | (0.39;7.01) | 0,46 | (0.05;4.32) |  | 3,17 | (0.67;14.93) | 2,46 | (0.64;9.43) |
| **Prior lifetime mental** | PTSD† | **121,20** | **(52.55;279.55)** ᶧ | **199,17** | **(41.2;962.85)** ᶧ |  | 2,01 | (0.65;6.17) | 0,30 | (0.05;1.82) |  | **3,96** | **(1.55;10.16)** ᶧ | 1,72 | (0.58;5.10) |
| **disorders** ‡ | Any other anxiety disorder | **2,52** | **(1.62;3.93)** ᶧ | 1,31 | (0.55;3.14) |  | **95,54** | **(36.84;247.77)** ᶧ | **133,85** | **(49.93;358.79) ᶧ** |  | **2,49** | **(1.81;3.43)** ᶧ | 1,31 | (0.9;1.92) |
|  | Any mood disorders | **4,47** | **(2.4;8.36)** ᶧ | 2,35 | (0.97;5.68) |  | **2,04** | **(1.37;3.03)** ᶧ | 0,43 | (0.23;0.82) |  | **20,95** | **(13.05;33.62)** ᶧ | **20,29** | **(13.05;31.53)** ᶧ |
|  | Any impulse/control disorder | **4,53** | **(1.41;14.58)** ᶧ | **28,25** | **(3.63;219.83)** ᶧ |  | 1,57 | (0.68;3.65) | 1,69 | (0.31;9.28) |  | 1,46 | (0.45;4.72) | 1,01 | (0.36;2.80) |
|  | Any substance disorders | 1,12 | (0.90;1.40) | 0,59 | (0.10;3.46) |  | 0,97 | (0.76;1.25) | 0,57 | (0.19;1.69) |  | 0,73 | (0.37;1.44) | 0,54 | (0.30;1.00) |
| **Earthquake's** | Rest of Murcia | 1 | - | 1 | - |  | 1 | 1 | 1 | - |  | 1 | - | 1 | - |
| **area** | Lorca | **7,09** | **(2.57;19.55)** ᶧ | 0,04 | (0.00;14.77) |  | 0,55 | (0.41;0.73) | 0,13 | (0.04;0.46) |  | 0,65 | (0.28;1.50) | 0,09 | (0.05;0.15) |
| **Global Earthquake’s Stressor Score (GESS)** | | **1,80** | **(1.45;2.23)** ᶧ | **4,06** | **(3.29;5.01)** ᶧ |  | 0,78 | (0.69;0.87) | 0,56 | (0.32;0.99) |  | 0,92 | (0.74;1.15) | **1,34** | **(1.01;1.77)** ᶧ |
| **Eathquake’s Experienced Stress (EES)** | | **1,30** | **(1.17;1.46)** ᶧ | 1,12 | (0.71;1.76) |  | **0,96** | **(0.93;1.00) ᶧ** | 1,43 | (1.00;2.05) ᶧ |  | 0,96 | (0.90;1.04) | **1,12** | **(1.05;1.19)** ᶧ |

**ᶧ** p-valor < 0.05; ^&^ Adjusted Odds ratio (OR) and 95%Confidence Interval (95%CI): all the variables in the table are included in the model. The variables GESS and EES are zero for the rest of Murcia and have been considered as continuous variables. All the estimates have been obtained using the part 2 weights. **‡** Prior lifetime mental disorders to earthquakes. **†** PTSD: Post-traumatic Stress Disorder;
